# Purification and Characterization of Schwann Cells from Adult Human Skin and Nerve

**DOI:** 10.1523/ENEURO.0307-16.2017

**Published:** 2017-05-16

**Authors:** Jo Anne Stratton, Ranjan Kumar, Sarthak Sinha, Prajay Shah, Morgan Stykel, Yuval Shapira, Rajiv Midha, Jeff Biernaskie

**Affiliations:** 1 Hotchkiss Brain Institute, University of Calgary, Calgary, Alberta T2N 4N1, Canada; 2 Department of Comparative Biology and Experimental Medicine Faculty of Veterinary Medicine, University of Calgary, Calgary, Alberta T2N 4N1, Canada; 3 Alberta Children’s Hospital Research Institute, University of Calgary, Calgary, Alberta T2N 4N1, Canada; 4 Department of Clinical Neurosciences Cumming School of Medicine, University of Calgary, Calgary, Alberta T2N 4N1, Canada

**Keywords:** autologous, axonal growth, nerve regeneration, remyelination, Schwann

## Abstract

Despite its modest capacity for regeneration, peripheral nervous system injury often results in significant long-term disability. Supplementing peripheral nervous system injury with autologous Schwann cells (SCs) may serve to rejuvenate the postinjury environment to enhance regeneration and ultimately improve functional outcomes. However, human nerve-derived SC (hN-SC) collection procedures require invasive surgical resection. Here, we describe the characterization of SCs from adult human skin (hSk-SCs) of four male donors ranging between 27 and 46 years old. Within five weeks of isolating and culturing adherent mixed skin cells, we were able to obtain 3–5 million purified SCs. We found that hSk-SCs appeared transcriptionally indistinguishable from hN-SCs with both populations exhibiting expression of SC genes including: *SOX10*, *SOX9*, *AP2A1*, *CDH19*, *EGR1*, *ETV5*, *PAX3*, *SOX2*, *CX32*, *DHH*, *NECL4*, *NFATC4*, *POU3F1*, *S100B*, and *YY1*. Phenotypic analysis of hSk-SCs and hN-SCs cultures revealed highly enriched populations of SCs indicated by the high percentage of NES^+ve^, SOX10^+ve^, s100^+ve^ and p75^+ve^ cells, as well as the expression of a battery of other SC-associated proteins (PAX3, CDH19, ETV5, SOX2, POU3F1, S100B, EGR2, and YY1). We further show that both hSk-SCs and hN-SCs are capable of promoting axonal growth to similar degrees and that a subset of both associate with regenerating axons and form myelin following transplantation into the injured mouse sciatic nerve. Interestingly, although the majority of both hSk-SCs and hN-SCs maintained SOX10 immunoreactivity following transplant, only a subset of each activated the promyelinating factor, POU3F1, and were able to myelinate. Taken together, we demonstrate that adult hSk-SCs are genetically and phenotypically indistinguishable to hN-SCs.

## Significance Statement

This work demonstrates for the first time that Schwann cells (SCs) can be isolated from adult human skin. Skin-derived SCs maintain their growth promoting properties and closely resemble SCs derived from the sciatic nerve, thus representing a highly accessible source of autologous SCs for nervous system repair.

## Introduction

Nearly 3% of all traumatic injuries result in peripheral nerve damage (Noble et al., 1998). Although the peripheral nervous system retains some capacity for regeneration, in most patients, sensorimotor recovery is incomplete, which results in lifelong impairment. In humans, much of this regenerative insufficiency is due to the long distances that axons need to travel to reinnervate target tissues. As a consequence of these distances, specialized glia called Schwann cells (SCs), which interact with the microenvironment to provide trophic support and remyelination of regenerating axons, are deprived of axon-derived feedback, resulting in atrophy and progressive loss of their growth-promoting influence (Sulaiman and Gordon, 2000; 2009; Stratton and Shah, 2016). Indeed, experimentally induced deficiencies in SC function results in profound impairment of nerve regeneration, further highlighting their important role in supporting endogenous nerve repair (Arthur-Farraj et al., 2012). One way to rejuvenate the microenvironment within the chronically denervated distal nerve or to extend the “regenerative” milieu beyond the subacute postinjury period (and to better distribute these effects across the entire length of the nerve), would be to replenish the nerve distal to the injury site with competent, autologous SCs, capable of supporting growth and myelination of regenerating axons. Indeed, since the 1980s, several groups have demonstrated functional gains following SC transplant in experimental models of spinal cord and nerve injury (Blakemore and Crang, 1985; Levi and Bunge, 1994a; Blakemore and Franklin, 1999). As a result of this work, further optimization studies are underway (Mirfeizi et al., 2017) as well as Phase I clinical trials (Saberi et al., 2011; Zhou et al., 2012; Guest et al., 2013).

Although promising, current SC collection procedures for autologous transplant require invasive surgical nerve resection, resulting in additional neurologic impairment and prolonged recovery times (Hilton et al., 2007). An alternative approach might be to source SCs from a more accessible source, such as the skin (Wong et al., 2006; Biernaskie et al., 2006). *Bona fide* SCs can be differentiated *in vitro* from isolated, self-renewing skin-derived precursors (SKPs) and following transplantation have been shown to improve anatomic measures such as axon growth, myelin clearance, modulation of endogenous glial cell behavior, and remyelination, ultimately supporting improved behavioral outcomes following spinal cord and nerve injury (McKenzie et al., 2006; Biernaskie et al., 2007; Walsh et al., 2009; Walsh et al., 2010; Khuong et al., 2014; Sparling et al., 2015; Kumar et al., 2016; Stratton et al., 2016). Although the majority of these studies have been done only using rodent cells, recent work shows myelination by human skin-derived SCs (hSk-SCs) is possible from the foreskin of neonatal children (Krause et al., 2014). Indeed, clinical relevance and potential will only be realized if SCs can be reliably derived from adult human skin (Wong et al., 2006) and, more importantly, that their functionality can be demonstrated.

To this end, we have isolated SCs from adult human skin samples using a rapid purification procedure that does not require an intermediate sphere formation step. Using a battery of SC lineage-specific markers, we demonstrate that adult hSk-SCs are phenotypically indistinguishable from human sciatic nerve-derived (hN-SCs) obtained from the same patient and are able to promote axon growth *in vitro* and, to some extent, initiate myelination of peripheral axons after murine nerve injury *in vivo*.

## Materials and Methods

### Adult human cell isolation, *in vitro* processing, and cell maintenance

#### Skin-derived SCs

hSk-SCs were collected from full thickness thigh skin obtained from four male autopsy donors (27, 28, 37, and 46 years of age) donated via the Southern Alberta Tissue Recovery and Transplant Program, under an approved protocol by the Conjoint Health Research Ethics Board at the University of Calgary. Skin was processed using similar techniques as previously described for human SKP cells (Toma et al., 2005; Biernaskie et al., 2006; Xie et al., 2008). Briefly, fat and blood vessels were removed from a 2-cm^2^ ellipse of skin, a total skin area deemed clinically relevant. This skin was then cut into small cubes and digested in dispase (5 mg/ml) for 2–3 h at 37°C. Epidermis was discarded and the dermis was minced and subsequently digested in collagenase Type IV (2 mg/ml) for 2–3 h at 37°C. DNase enzyme (40 ng/ml) was added, tissue triturated, and further incubated at 37°C for 1–2 h. Samples were then centrifuged (300 × *g* for 5 min) and then resuspended in DMEM. Cell counts were determined and cells cultured at a density of 10,000 cells/ml on poly-D-lysine (20 μg/ml) and laminin (4 μg/ml)-coated surfaces (BD Bioscience). Cells were cultured in DMEM and F12 (3:1) with neuregulin (50 ng/ml), forskolin (5 μM), N2 supplement (1%), and penicillin (100 μg/ml)/streptomycin (1%, Invitrogen; herein referred to as SC media) at 37°C in a 5% CO_2_ incubator. To prevent mycoplasma/fungus contamination all cell cultures were supplemented with plasmocin (25 μg/mL, Invitrogen) and fungizone (40 ng/ml, Invitrogen) and tested routinely for mycoplasma using PCR. Triple-filtered fetal bovine serum (1% FBS; Hyclone) was also added for the first 5 d to improve cell survival. After three to four weeks, bipolar SC colonies were identified, marked and isolated using cloning cylinders, as described previously (Biernaskie et al., 2006). Sterile cloning cylinders (Corning) coated with vacuum grease were placed over a colony and pressed down to form a seal. Cylinders were then filled with trypLE-express and incubated at 37°C for 5 min. All detached cells within the cylinder were collected and plated at a density of 50,000 cells/ml for further expansion.

#### Nerve-derived SCs

Human sciatic nerve samples were obtained from the same four donors as noted above. SCs were isolated using a modified protocol from previously described methods (Morrissey et al., 1991; Rutkowski et al., 1992; Rutkowski et al., 1995; Haastert et al., 2007). Briefly, individual nerve fascicles were dissected and minced, and subsequently incubated in collagenase Type IV (Worthington) at 37°C for 1 h triturating every 20 min. Samples were then centrifuged (1000 rpm for 5 min), resuspended in DMEM, counted, and then resuspended in SC media as described above. All skin and nerve cells were fed every fourth day and treated identically.

#### Fibroblasts

Human skin-derived fibroblasts were harvested using the same methods as described above. Importantly, media contained 10% FBS instead of neuregulin and forskolin which allowed fibroblasts in our mixed dermal cultures to outcompete all other cells types within these dermal cultures. All fibroblast cultures were treated in the same fashion as above.

### Lentiviral transduction and FACS/flow

To track the fate of donor cells following *in vivo* transplantation, adult human cells were transduced with GFP-expressing lentiviral particles as done previously (Rahmani et al., 2013). All virus work was undertaken in a biohazard level 2 laboratory using previously established protocols (Hotta et al., 2009). Cells were grown to 40% confluence and then incubated overnight in OptiMEM medium supplemented with supernatant containing GFP-expressing lentiviral particles (under EF1α promoter control) and polybrene (8 μg/ml). After 18 h, cells were then washed, and media were then replaced with SC growth medium. Cells were then dissociated and FACS sorted (BD FACS Aria III) to further purify the GFP+ cell fraction (∼40–70% of cells were GFP-positive). For flow analysis, cells were dissociated then blocked in 2% BSA, before primary antibody (p75; Table 1) was added for a 20-min incubation at room temperature. Unbound primary antibodies were washed then cells were incubated with Alexa Fluor-conjugated secondary antibodies at room temperature for 20 min before performing flow analysis. No primary controls were used to set gates.

**Table 1.** Primary antibody details for immunocytochemistry and immunohistochemistry

Primary antibody	Type	Raised in	Working dilution	Source	RRID	Catalog number
Anti-YY1	Monoclonal	Rabbit	1:50	Cell Signalling Technology	RRID:AB_823672	2185
Anti-Sox2	Polyclonal	Goat	1:100	Santa Cruz Biotechnology	RRID:AB_2286684	17320
Anti-S100	Polyclonal	Rabbit	1:500	Dako	RRID:AB_10013383	Z0311
Anti-S100β chain	Polyclonal	Rabbit	1:200	Santa Cruz Biotechnology	RRID:AB_2184560	28533
Anti-Nav1.6 (scn8a)	Polyclonal	Rabbit	1:100	Millipore	RRID:AB_2314858	ab5580
Anti-Pax3	Monoclonal	Rabbit	1:50	Cell Signalling Technology	RRID:AB_2636922	12412
Anti-PZero Myelin Protein	Polyclonal	Chicken	1:500	Aves Lab	RRID:AB_2313561	PZO
Anti-Oct6 (POU3F1)	Polyclonal	Goat	1:50	Santa Cruz Biotechnology	RRID:AB_2268536	11661
Anti-Neuronal class β-III tubulin/Tuj1	Monoclonal	Mouse	1:1000	Covance	RRID:AB_2313773	MMS-435P
Anti-Ki67, clone Sola15	Monoclonal	Rat	1:500	eBiosciences	RRID:AB_10854564	14569882
Anti-NES	Monoclonal	Mouse	1:500	Cell Signalling Technology	RRID:AB_2235913	4760
Anti-Myelin basic protein	Monoclonal	Rat	1:500	Millipore	RRID:AB_240845	MAB395
Anti-Krox20/Egr-2	Polyclonal	Rabbit	1:50	Covance	RRID:AB_10064079	PRB-236P
Anti-Sox10	Polyclonal	Goat	1:200	Santa Cruz Biotechnology	RRID:AB_2255319	17343
Anti-Etv5	Monoclonal	Mouse	1:100	R&D Systems	RRID:AB_10997135	MAB7107
Anti-GFP	Polyclonal	Chicken	1:500	Millipore	RRID:AB_310288	06896
Anti-Fibronectin	Polyclonal	Mouse	1:200	BD Biosciences	RRID:AB_397486	610078
Anti-Cadherin-19	Polyclonal	Rabbit	1:50	Santa Cruz Biotechnology	RRID:AB_2077401	84771
Anti-P75	Polyclonal	Rabbit	1:100	Promega	RRID:AB_430853	G323
Anti-Caspr, clone K65/35	Monoclonal	Mouse	1:40	NeuroMab	RRID:AB_10671175	73-001
Anti-CNTF	Polyclonal	Goat	1:100	R&D Systems	RRID:AB_354368	ab557na

### Karyotyping and authentication

To assess chromosome integrity, karyotyping was performed using previously described methods (Fan et al., 2013). Cultures (∼75% confluent) were treated with KaryoMAX Colcemid solution (30 ng/mL, Gibco) for 4 h at 37°C. SCs were then detached from dishes, centrifuged, then resuspended in cell hypotonic solution (40 mM KCl, 20 mM HEPES, 0.5 mM EGTA, and 9 mM NaOH) and incubated for 1 h at 37°C. After 1 h, the mix was fixed with acetic acid/methanol (1:3), and G-banding analysis was performed in the Clinical Genetics Facility at the Alberta Children’s Hospital. We performed third party authentication (TCAG, Sick Kids Research Institute), for human cell origin and absence of rodent DNA to ensure purity of donor human cells before transplant. We cultured cells as above, detached from dishes, centrifuged and then extracted DNA (QIAGEN kit). DNA was of high quality and purity (260/280 and 260/230 ratios of ∼1.8; 10–20 ng/μl). We performed PCR against the Ogg1 gene that is predicted to exclusively amplify rodent, and not human DNA (primers: ACTGCATCTGCTTAATGGCC CAGCATAAGGTCCCCACAGA). Indeed, rodent-specific bands were not detected in human cell samples (data not shown).

### Immunocytochemistry

Adult human SCs and fibroblasts were grown to 80% confluence, washed with sterile PBS and then fixed in 4% paraformaldehyde (PFA) for 5 min. Cells were then permeabilized using 0.5% Triton X-100 and 5% BSA for 1 h at room temperature. Primary antibodies (Table 1) were incubated at 4°C overnight. Anti-Nestin (NES) (for SC cultures) or anti-FN1 (for fibroblast cultures) antibodies were used in all cases, as a costain to validate within each culture that the appropriate cell type of interest was indeed present. The next day, unbound primary antibodies were washed then incubated with Alexa Fluor-conjugated secondary antibodies at room temperature for 1 h or/and Fluoromyelin (1:200, Life Technologies), washed, and nuclei were counterstained with Hoechst (1:2000, Sigma) and mounted with Permafluor mounting media (for confocal imaging) or left in PBS (for ImageExpress imaging). For quantification, cells were imaged and quantified using an ImageXpress Microscope (Molecular Devices). Each condition consisted of cells from three to four donors, with three to four technical replicates (consisting of 12 images/replicate), per cell type per antibody.

### Quantitative gene expression analysis

To assess the genetic profile of adult human SCs and fibroblasts, cells were allowed to grow in SC media until 80% confluent, or in differentiation/promyelination media (50 μg/ml ascorbic acid, 0.5 μM forskolin, 5 ng/ml neuregulins, and 5 ng/ml NGF) for 14 d on axons grown as previously described (Kumar et al., 2016). They were then washed with sterile PBS and 500,000 cells were pelleted, and snap frozen. mRNA from cell pellets was extracted using a Dynabeads DIRECT kit, according to manufacturer’s guidelines. RNA quantity and quality were assessed by Tapestation and Qubit QC. Sixty-five human SC-specific and two fibroblast-associated probe sequences were incorporated into custom designed nCounter codesets (Nanostring Technologies). Each CodeSet included seven housekeeping genes to correct for RNA amount and ensure quality. Data were subjected to an internal QC process and from seven candidate housekeeping genes, Rpl13a was selected as being most appropriate based on its consistency across samples. A total of 100 ng of total RNA for each reaction was used. The analysis was done according to manufacturer instructions. Briefly, raw counts were first background adjusted with a truncated poisson correction using internal negative controls was and then log2 transformed. Data were then corrected for input amount variation through a sigmoid shrunken slope normalization step using the mean expression of housekeeping genes. Each condition consisted of nerve and skin-derived SCs from four different human donors with statistical analyses performed on the mean fold-change of nerve versus skin-derived SCs after normalizing to patient-matched fibroblast controls.

### Neuronal explant cultures

For *in vitro* neurite outgrowth experiments, neonatal Sprague Dawley rats (postnatal day 2, *n* = 12) were used to obtain dorsal root ganglia (DRG) explants. DRG explants were isolated then grown on poly-D-lysine, matrigel, and laminin-coated chamber slides (Fisher Scientific) in DRG growth media [DMEM:F12; 3:1, containing nerve growth factor (NGF; 50 ng/ml), FBS (20%), penicillin/streptomycin (1%), and B27 supplement (2%)]. To eliminate endogenous host SCs, all DRGs were treated with cytosine arabinoside (Ara-C; 7 μM) for 4 d and then human SC-conditioned media were added in the presence of 1% FBS and 5 ng/ml NGF to determine whether human SCs provide paracrine promotion of axonal growth. DRGs were grown in conditioned medium under three different conditions: (1) control, where SC growth media were kept in incubator for 5 d in the absence of cells, before plating onto DRGs; (2) hSk-SC, where conditioned media from human skin SCs (passage 5–7) from our three human samples collected after 5 d in culture at 75% confluence, before plating onto DRGs; and (3) hN-SC, where conditioned media from human nerve SCs (passage 5–7) from our three human samples collected after 5 d in culture at 75% confluency. DRGS were grown for 7 d in conditioned media (with one media change at day 4), then fixed with 4% PFA. Neurites were immunostained with neuronal class β-III tubulin and imaged on an SP8 spectral confocal microscope. Using ImageJ software, concentric rings were overlayed (200 µm apart) and the number of neurites intersecting each concentric circle was quantified. For each condition, seven to eight explants were assessed.

### Cell transplantation

All experimental procedures involving animals received prior approval from the University of Calgary Animal Care Committee and were in accordance with guidelines provided by the Canadian Council for Animal Care. For transplants into peripheral nerve sciatic crush injury, adult immune-deficient NOD/CB17-*Prkdc^scid^*/NcrCrl mice (*scid* mice) were used as donor cell recipients following injury (8–10 weeks old, male, *n* = 30, Charles River). All rodents were kept in a 12/12 h light/dark cycle, temperature-controlled environment with unlimited food and water. All procedures were done as previously described (McKenzie et al., 2006; Kumar et al., 2016). Briefly, sciatic nerves were exposed, and a mid-thigh level crush was induced using number 5 surgical forceps for 60 s while mice were under deep anesthetic (2% isoflurane). Using a 33-gauge microsyringe (Hamilton), each injured nerve (distal to the crush site) received a 2-µl volume suspension containing 100,000 cells. Cell suspensions contained either: adult human GFP^+ve^ nerve-derived SCs (*n* = 3 mice per donor, four donors), adult human GFP^+ve^ skin-derived SCs (*n* = 3 mice per donor, four donors) or carrier media alone (*n* = 6). SC suspensions also contained neuregulin (500 ng/ml) and fast green (1%) in DMEM to enhance cell survival and to identify successful injections into the nerve fascicle. Animals received buprenorphine analgesia (0.05–0.1 mg/ml) for up to 5 d after surgery. Immunohistochemistry and microscopic imaging. Sciatic nerves were harvested at five to eight weeks after injury, then fixed for 2 h with 2–4% PFA. All nerves were submerged in 30% sucrose overnight. Tissue was then frozen in OCT (VWR International) and stored at −80°C. Sections were cut using a Leica cryotome at 10–18 µm. For immunofluorescence staining, sections were thawed at room temperature, permeabilized with 0.5% Triton X-100 and blocked with BSA (5%). Primary antibodies (Table 1) were incubated overnight and secondary goat or donkey Alexa Fluor-conjugated antibodies (Invitrogen, 1:200) and/or Fluoromyelin (F34652, Life Technologies) were applied for 2 h at room temperature. Hoechst was used to stain nuclei (1:1000, Sigma), then slides were mounted with Permafluor (Thermo Fisher Scientific). Image collection and quantification was done using a Leica SP8 confocal microscope. Images from each nerve were collected using a 63× objective lens and Z-stack (eight planes) features. Images (maximum projection or orthogonal view) were analyzed using ImageJ (NIH). Donor cell myelination was defined as GFP^+ve^ myelinating cells that were associated with MBP^+ve^ myelin (as shown in representative images). Cell fate quantification was done by counting the total number of GFP^+ve^ cells relative to the total number of GFP^+ve^ cells that coexpressed each marker of interest (SOX10, POU3F1, or MBP). Counts were done from two to three images per animal systematically selected across the thickness of the injured nerve. Counts were then expressed as a ratio (i.e., GFP^+ve^ MBP^+ve^ cells/GFP^+ve^ cells) and averaged within each animal (*n* = 3 animals/group, three donors per group).

### Immunogold electron microscopy

Sciatic nerves were harvested at eight weeks after injury, then fixed overnight with 4% PFA. Fixed samples were then placed in sucrose (2.3 M) in PBS overnight. Freeze substitution was then conducted using a Leica AFS. Samples were freeze substituted in 0.2% uranyl acetate in methanol for 48 h at −90°C. The temperature was increased to −35°C over 18 h. The samples were washed 4× 15 min with 100% methanol, then infiltrated with Lowicryl HM20 resin as follows: 1:1 HM20:methanol overnight, 3:1 HM20:methanol 3 h, 100% HM20 3 h, 100% HM20 overnight. The samples were placed in molds filled with fresh HM20, and the resin was polymerized using UV light for 48 h. Thin sections were cut and picked up on Formvar-coated nickel grids before immunolabelling was performed. Grids were placed on drops of 0.15 M glycine in PBS for 10 min, then 1% PBS-c (Aurion) in PBS for 30 min, then primary antibody (rabbit anti-GFP) diluted 1:20 in 0.1% BSA-c in PBS for 2 h. Grids were then washed 6× 5 min with PBS. Secondary antibody (donkey anti- rabbit IgG conjugated to 12 nm colloidal gold; Jackson Laboratories) diluted 1:20 in 0.1% BSA-c in PBS was then added for 1.5 h, then washed for 6× 5 min with PBS. Sections were then fixed with 2% glutaraldehyde in 0.1 M Sorenson’s phosphate buffer (pH 7.4), then washed 2× 5 min with PBS followed by 3× 5 min with dH_2_O. Sections were stained with uranyl acetate and lead citrate. Sections were viewed using a Hitachi H7500 transmission electron microscope and images captured using an Olympus SIS Megaview II 1.35 MB digital camera and iTEM version 5.2 software.

### Statistical analysis

All statistical analysis was undertaken using GraphPad Prism (v5.0). Statistical comparisons with a single variable were done using a Student’s *t* test or one-way ANOVA followed by Tukey’s *post hoc* test and *p* < 0.05 was considered statistically significant. All graphs are presented as mean ± SEM.

## Results

### SCs isolated directly from adult human skin can be reliably purified in three to five weeks and retain normal chromosomal and SC features

**Figure 1.**
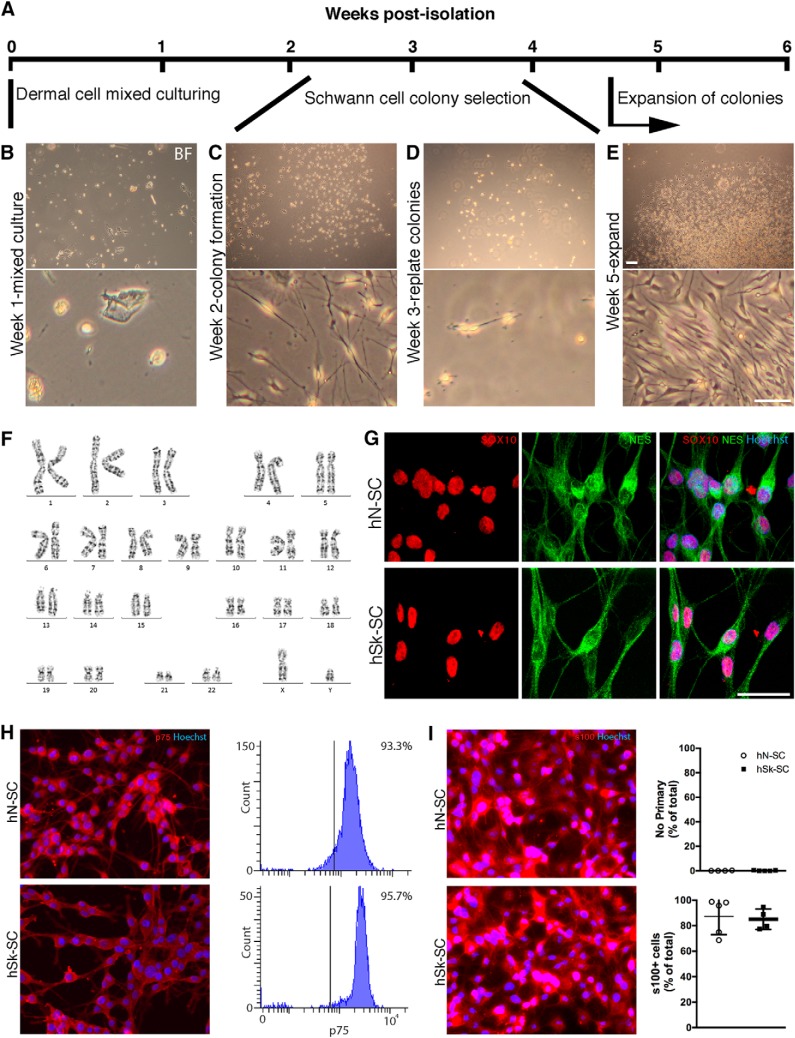
Rapid purification and *in vitro* processing of adult hSk-SCs does not alter basic SC characteristics. ***A***, Timeline of isolation, purification, and expansion of skin-derived SCs. ***B–E***, Representative bright field images of skin-derived SCs during the initial stages of isolation, purification, and expansion. ***F***, Chromosomes isolated from a representative adult hSk-SC that had been previously passaged and expanded *in vitro* for five weeks. ***G–I***, Representative immunocytochemical images and quantification of adult human nerve (hN-SC)- and skin (hSk-SC)**-**derived SCs revealed that the majority of cells in culture expressed SOX10, NES, s100, and p75. Scale bar, 25 μm. All representative images are of cells derived from a 46-year-old male.

Using approaches described in Materials and Methods, we were able to isolate and expand SCs directly from a clinically relevant sized sample of skin from all four patients (Fig. 1*A*,*B*). Within two weeks of culturing adherent skin cells in SC media, colonies of bipolar shaped cells were detectable (Fig. 1*C*). Colonies could be selected using cloning cylinders, replated (Fig. 1*D*), and allowed to expand for several weeks (Fig. 1*E*). By five weeks, we had obtained 3–5 million purified SCs. To ensure that cells isolated and expanded from adult human skin autopsies, in this fashion, maintained normal features, we assessed the number and integrity of chromosomes after several weeks of expansion. We found that cells maintained normal chromosomal features, including normal G-banding (Fig. 1*F*). We also found that the majority of cells expressed SC-associated markers (i.e., NES, SOX10, s100, p75) consistent with a SC identity. Immunocytochemical and flow quantification revealed that the percentage of skin-derived cells versus nerve-derived SCs (from the same patient) that expressed NES, SOX10, s100, and p75 was indistinguishable [88.4 ***±***5.0% and 94.9 ***±***1.6% of total Hoechst^+^ cells expressed NES (*p* = 0.3, immunocytochemistry), 89.3 ***±***6.3% and 77.3 ***±***6.2% of total Hoechst^+^ cells expressed SOX10 (*p* = 0.2, immunocytochemistry), 85.1 ***±***4.0% and 87.3 ***±***6.4% of total Hoechst^+^ cells expressed s100 (*p* = 0.8, immunocytochemistry), and 93.3% and 95.7% of cells expressed p75 (*p* = 0.4, flow) in hN-SCs vs hSk-SCs, respectively; unpaired *t* test; Fig. 1]. We also found that the percentage of Fibronectin+ fibroblasts was minimal and equivalent in cultures from either group (10.3 ± 5.1% in hN-SCs vs hSk-SCs 5.4 ± 5.3% cultures; *p* = 0.5; unpaired *t* test).

### Adult human nerve and skin-derived SCs are genetically indistinguishable and express genes associated with several distinct stages of SC development

**Figure 2.**
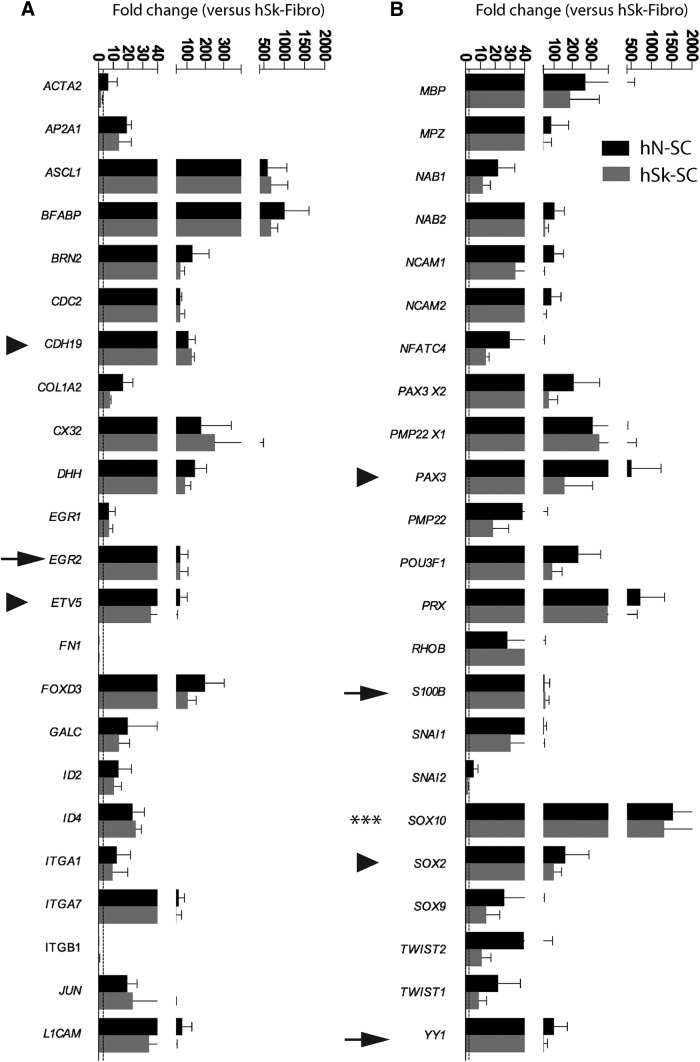
Adult human nerve and skin-derived SCs are genetically indistinguishable *in vitro*, and express genes associated with several distinct stages of SC development. Quantification of mRNA levels in cultured adult human nerve (hN-SC)- and skin (hSk-SC)-derived SCs, cultured in identical conditions revealed that both hN-SCs and hSk-SCs expressed genes associated with several distinct stages of SC development at similar levels. Arrowheads represent a selection of precursor or immature associated genes. Arrows represent a selection of mature or promyelinating-associated genes. Asterisks represent a gene associated with all stages of SC development. Each bar represents the mean of three patient SC cultures (27-, 37-, and 46-year-old male) normalized against a housekeeping gene, and then expressed against the mean expression of cultures of adult human dermal fibroblasts (mean ± SEM, unpaired *t* test).

To compare the genetic profiles of adult human nerve and skin-derived SCs, we cultured these cells under identical conditions, then compared the transcript levels for a battery of SC-associated genes. We found that several SC-associated genes were highly expressed in both nerve and skin-derived SC cultures with an average fold increase of 98.7 ± 23.0 and 146 ± 28.7, respectively, across all genes assessed, compared with dermal fibroblast cultures (Fig. 2). This included genes that exhibit sustained expression across all stages of SC development (*SOX10*, *SOX9*), or genes enriched in immature or SC precursors (*AP2A1*, *CDH19*, *EGR1*, *ETV5*, *PAX3*, and *SOX2*) or promyelinating SCs (*CX32*, *DHH*, *EGR2*, *MBP*, *NECL4*, *NFATC4*, *PMP22*, *POU3F1*, *S100B*, and *YY1*; Jung et al., 2015; Balakrishnan et al., 2016). Importantly, there was no difference in the expression levels between skin and nerve-derived SC cultures, of all genes that were assessed (*p* > 0.05, repeated unpaired *t* test). As expected, there was reduced expression of genes associated with fibroblasts (*FN1, ITGB1*) in both nerve and skin-derived SC cultures compared with fibroblast cultures (hN-SC: −3.8 ± 1.9-fold; hSk-SC: −4.9 ± 0.15-fold).

### Adult human nerve and skin-derived SCs are phenotypically indistinguishable *in vitro*, and express proteins associated with several distinct stages of SC development

**Figure 3.**
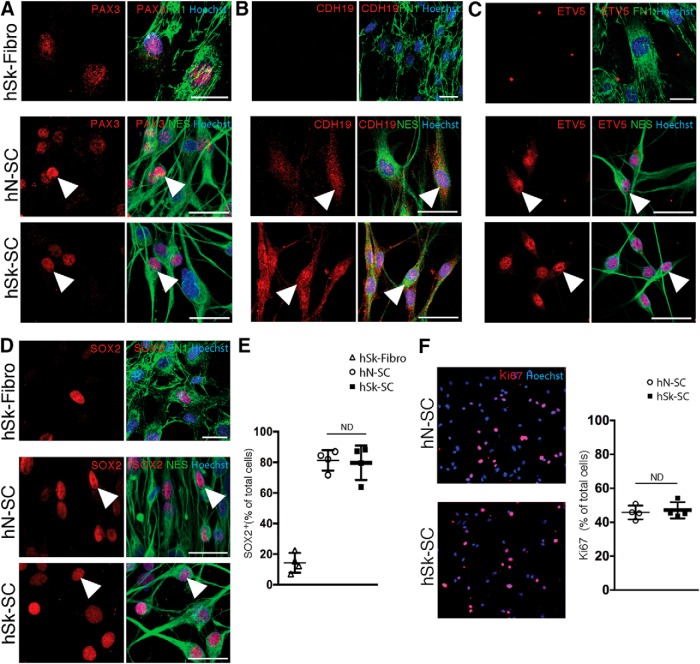
Adult human nerve- and skin-derived SCs are phenotypically indistinguishable *in vitro* and express proteins associated with precursor and immature stages of SC development. ***A–D***, Representative immunocytochemical confocal images (46-year-old male) of adult human nerve (hN-SC)- and skin (hSk-SC)**-**derived SCs (NES^+^, green) and adult human skin-derived dermal fibroblasts (hSk-Fibro, FN1^+^, green) cultured in identical conditions. Immunocytochemistry revealed that SCs isolated from both human skin and nerve express several precursor and immature SC antigens (red, arrowheads), including (***A***) PAX3, (***B***) CDH19, (***C***) ETV5, and (***D***) SOX2. ***E***, Quantification of the percentage of Hoechst^+^ cells expressing SOX2 in SC cultures. ***F***, hSk-SCs also expressed the proliferative marker, ki67, in a similar percentage of cells to hN-SCs. Each dot represents the mean percentage within a patient culture (27-, 28-, 37-, and 46-year-old male**)** across three to four replicates (mean ± SEM, unpaired *t* test; scale bar, 25 μm).

**Figure 4.**
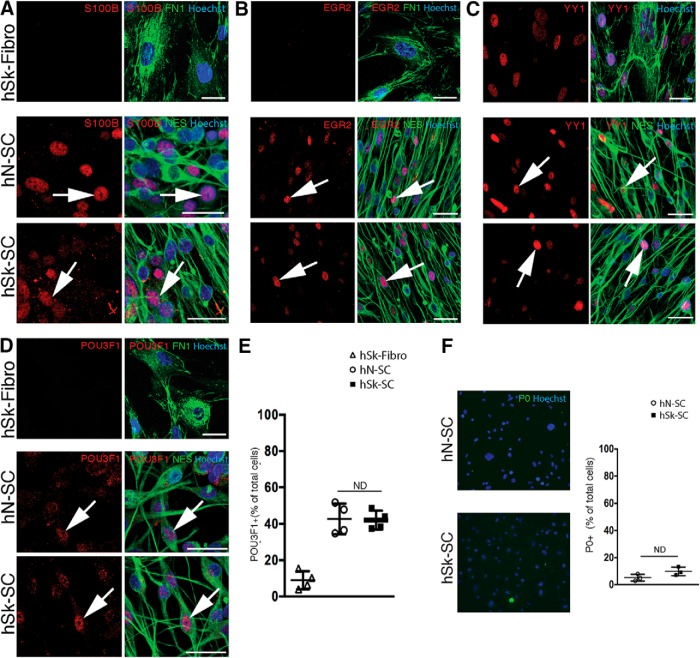
Adult human nerve and skin-derived SCs are phenotypically indistinguishable *in vitro* and express proteins associated with promyelinating and mature myelinating stages of SC development. Representative immunocytochemical confocal images (46-year-old male) of adult human nerve (hN-SC)- and skin (hSk-SC)**-**derived SCs (NES^+^, green) and adult human skin-derived dermal fibroblasts (hSk-Fibro, FN1^+^, green) cultured in identical conditions. Immunocytochemistry revealed that SCs isolated from both human skin and nerve express several promyelinating and mature myelinating SC antigens (red, arrows), including (***A***) S100B, (***B***) EGR2, (***C***) YY1, (***D***) POU3F1. ***E***, Quantification of the percentage of Hoechst^+^ cells expressing POU3F1 in individual patient SC cultures. ***F***, hSk-SCs also did not express the mature myelin protein, P0, comparable to hN-SCs. Each dot represents the mean percentage within a patient culture (27-, 28-, 37-, and 46-year-old male**)** across three to four replicates (mean ± SEM, unpaired *t* test; scale bar, 25 μm).

Under identical conditions, as above, we performed immunostaining for SC-associated proteins. Using NES as a reliable marker of SCs *in vitro* (Kumar et al., 2016), we performed coexpression analysis of several additional proteins in nerve and skin-derived SC cultures as well as patient-matched dermal fibroblast cultures (Figs. 3, 4). This included proteins known to be associated with defined stages, such as SC precursor/immature SC stages [PAX3 (Fig. 3*A*), CDH19 (Fig. 3*B*), ETV5 (Fig. 3*C*), and SOX2 (Fig. 3*D*)] or promyelinating/myelinating SC stages [S100B (Fig. 4*A*), EGR2 (Fig. 4*B*), YY1 (Fig. 4*C*), and POU3F1 (Fig. 4*D*)]. We found that the majority of both nerve and skin-derived SC cultures expressed SOX2 (79.69 ± 5.64% in hN-SCs and hSk-SCs 81.28 ± 3.34% cultures; *p* = 0.8, hN-SCs vs hSk-SCs: no difference, unpaired *t* test; Fig. 3*D*,*E*). A similar pattern was observed with PAX3, CDH19, and ETV5, all factors that are, like SOX2, associated with early SC development or the de-differentiated SC state (Fig. 3*A–C*). In contrast, proteins associated with later developmental stages were observed only in a subset of SCs in both nerve and skin cultures. For example, we found that 42.72 ± 4.17% and 42.09 ± 2.59% of cells in nerve and skin-derived SC cultures, respectively, expressed POU3F1 (*p* = 0.9, hN-SCs vs hSk-SCs: no difference, unpaired *t* test; Fig. 4*D*,*E*), and the percentage of myelin protein P0+ SCs was low and equivalent between groups (*p* = 0.1, 5.2 ± 1.5% in hN-SCs vs 9.9 ± 1.8% in hSk-SCs cultures; no difference, unpaired *t* test; Fig. 4*F*). A similar pattern was observed with S100B, EGR2, and YY1, all factors that are generally associated with late SC development and initiation of a myelination program (Fig. 4*A–C*). Interestingly, the percentage of Ki67^+^ proliferating SCs was also equivalent between groups (47.09 ± 2.41% in hN-SCs vs hSk-SCs 45.74 ± 2.04% cultures; *p* = 0.7; Fig. 3*F*).

### Factors secreted from adult human nerve and skin-derived SCs promote neurite outgrowth *ex vivo*

**Figure 5.**
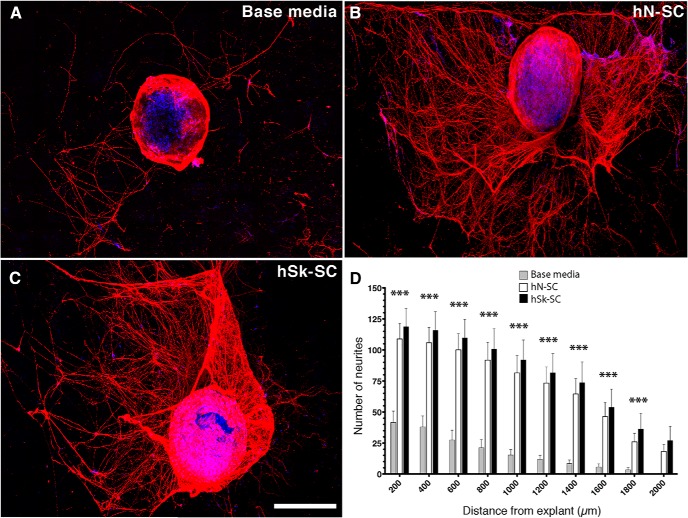
Nerve and skin-derived SCs produce growth factors that promote neurite outgrowth *ex vivo*. Representative immunohistochemical images of rat DRG explants immunostained for βIII tubulin (red) and Hoechst (blue). Explants were treated with (***A***) SC base media, (***B***) nerve-derived SC**-**conditioned media, or (***C***) skin-derived SC**-**conditioned media. ***D***, Quantification of the total number of neurites from 200 to 2000 μm from the explant. *n* = 7–8 individual explants per patient (*n* = 3–4 patient media per group). Data are shown as mean ± SEM. Data were analyzed with one-way ANOVA with Tukey *post hoc* test comparison to base SC media. ***p < 0.001. Scale bar, 500 µm.

To assess the capacity of adult human nerve and skin-derived SCs to promote axonal growth, we applied conditioned media from either population to DRG explant cultures (Varon et al., 1981; Bosch et al., 1988; Hu et al., 2011; Kumar et al., 2016; Fig. 5). Using digital concentric ring analysis, we quantified the number of intersecting DRG neurites up to 2 mm from the explant. We found significantly fewer intersecting neurites in base media conditions (Fig. 5*A*) compared with nerve (Fig. 5*B*) and skin-derived SC (Fig. 5*C*) conditions; 200 μm (41.63 ± 9.16 vs 118.71 ± 14.78, 98.50 ± 12.56) to 1800 μm (3.38 ± 1.81 vs 36.29 ± 12.74, 28.25 ± 6.94; *p* < 0.01; Fig. 5*D*) distances. Nerve versus skin-derived SC conditions did not differ at any point suggesting that hSk-SCs provide similar secreted trophic support for regenerating axons.

### Adult human nerve and skin-derived SCs are able to ensheath regenerating murine axons, upregulate myelin genes, and generate myelin with modest efficiency

**Figure 6.**
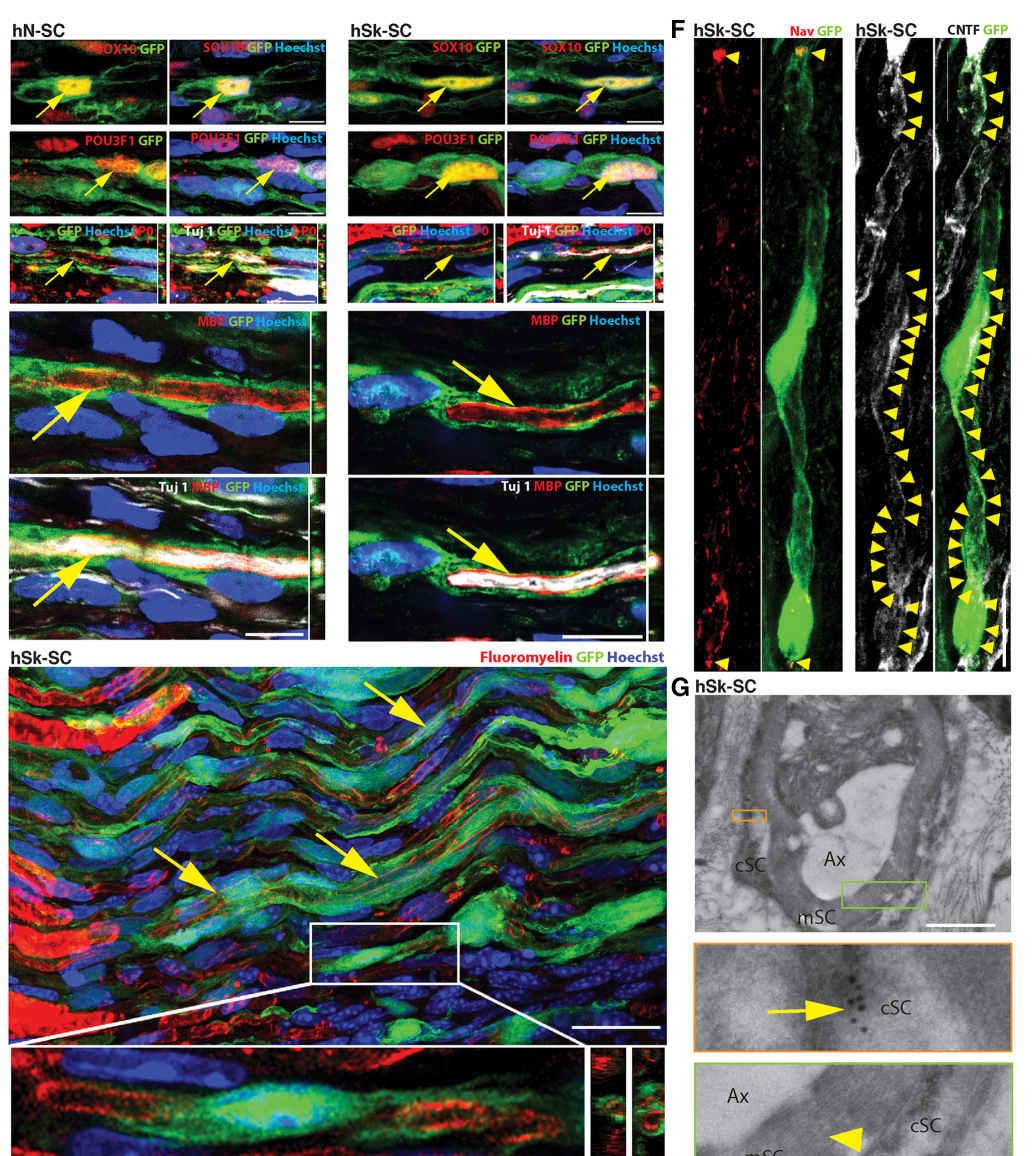
Adult human nerve and skin-derived SCs are able to ensheath rodent axons and produce limited myelin. SCs were transplanted into nerve injury and assessed by immunofluorescence at five to eight weeks after injury. ***A–F***, Representative images show GFP-positive adult human nerve (hN-SC) and skin (hSk-SC) SCs (green) coexpressing (red, arrows) the pan-SC protein, SOX10 (***A***), the promyelinating factor, POU3F1 (***B***), the myelin proteins, P-zero (***C***) and MBP (**D**), and the myelin lipid marker, Fluoromyelin (***E***). Insets show higher magnification image of SCs and Z-plane view of the close association of myelin proteins with GFP. We also noted the presence of Nav1.6^+^ sodium channels at the nodes (red, arrowheads; ***F***), and CNTF^+^ mesaxons throughout the internode (white, arrowheads; ***F***), as well as GFP^+^ immunogold particles (inset, arrow; ***G***) associated with compact myelin (inset, arrowhead; ***G***). Scale bar: 10 μm (***A–F***), 25 μm (***E***), 1 μm (***G***); Ax, axon; cSC, SC cytoplasm; mSC, myelin (***G***). Representative images are from 46- and 27-year-old male.

**Figure 7.**
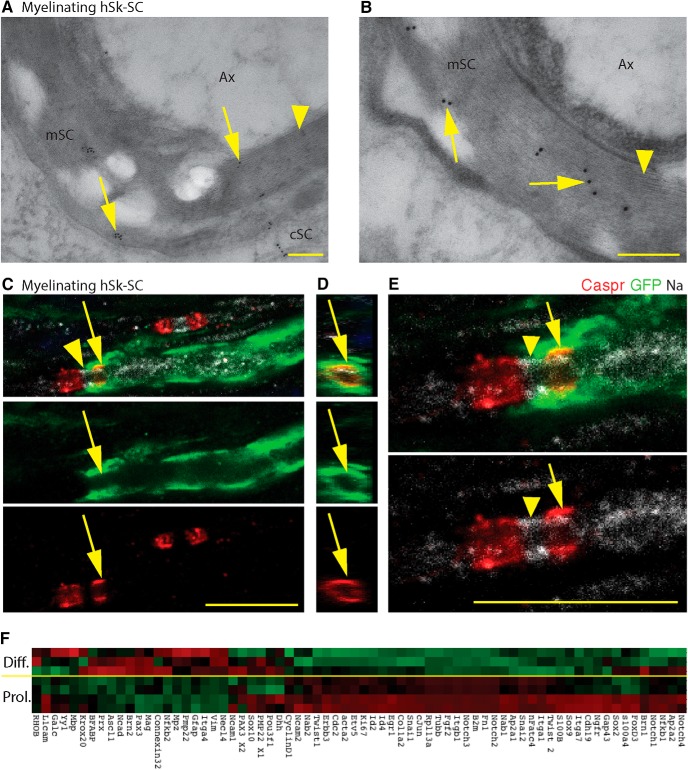
Adult hSk-SCs form compact myelin and induce axonal expression of paranodal/nodal proteins. ***A*, *B***, High-resolution representative electron microscopic images of GFP^+^ immunogold particles (arrows) associated with compact myelin (arrowheads). ***C–E***, High-resolution representative X-Y **(*C***, ***E*)** and Z-plane views (***D***) of an immunohistochemical image of a myelinating human SC (GFP, green) associated with paranodal (Caspr, red, arrow) and nodal (Na, Nav1.6+ sodium channels, white, arrowhead) proteins. ***F***, When hSk-SCs were added to neuronal explant cultures (Diff.), by 10 d after addition, these cells had upregulated a plethora of genes associated with myelination as well as downregulated genes associated with immature SCs in comparison to hSk-SCs maintained in standard SC growth conditions (Prol.). Red, high relative expression; green, low relative expression. Scale bars, 0.2 μm (***A***, ***B***) and 10 μm (***C–E***); hSk-SC, human skin-Schwann cell; Ax, axon; cSC, SC cytoplasm; mSC, myelin. Representative images are from 46- and 27-year-old male.

To compare the myelinating capacity of adult human nerve versus skin-derived SCs, we transplanted GFP-labeled human SCs into a sciatic nerve crush injury model. At eight weeks after transplant, the majority of GFP^ve+^ cells retained expression of SOX10 (Fig. 6*A*), in both nerve and skin-derived SC transplants (*p* = 0.3, 96.49 ± 1.28% vs 97.90 ± 0.52%: no difference, unpaired *t* test). We further assessed their phenotypic status demonstrating that 60.85 ± 2.21% vs 52.37 ± 1.21% of GFP^ve+^ cells expressed POU3F1 in nerve and skin-derived SCs, respectively (*p* = 0.2, hN-SCs vs hSk-SCs: no difference, unpaired *t* test; Fig. 6*B*). This was a 30-40% increase compared with what we observed *in vitro*, suggesting that both SC types appeared to respond to endogenous cues by activating a promyelinating gene. Assessment of the mature myelin-associated protein, MBP (Fig. 6*D*) demonstrated that 4.45 ± 1.56% and 3.07 ± 1.20% of GFP^ve+^ nerve and skin-derived SCs coexpressed MBP, aligning in parallel with neurofilament^+^ axons (*p* = 0.5, hN-SCs vs hSk-SCs: no difference, unpaired *t* test). P-zero, another myelin-associated protein (Fig. 6*C*) showed similar colabeling frequency in donor SCs of both origins. A thorough analysis of axon-associated GFP^ve+^ hSk-SCs revealed the presence of Fluoromyelin, a lipophilic myelin-binding dye (Wu et al., 2006; Fig. 6*E*), the presence of sodium channels at the node of Ranvier (Figs. 6*F*, 7), mesaxons throughout the internode (Fig. 6*F*), and Caspr proteins at the paranode (Fig. 7*C–E*). Electron microscopic ultrastructural analysis using immunogold labeling for GFP confirmed the presence of compact myelin associated with GFP^ve+^ skin-derived SCs (Figs. 6*G*, 7*A*,*B*). In addition, when hSk-SCs were added to neuronal explant cultures, by 10 d after addition, these cells had upregulated a plethora of genes associated with myelination as well as downregulated genes associated with immature SCs in comparison to hSk-SCs maintained in standard SC growth conditions (Fig. 7*F*).

## Discussion

We established that adult human SCs could be cultured directly from skin obtained from a clinically relevant sample size of adult human skin. Importantly, we demonstrated that our culturing approach allows for the purification and expansion of these cells without the need for previously described intermediate processing steps (Toma et al., 2005; Krause et al., 2014) or chronic exposure to animal serum (Levi et al., 1994b). We demonstrated that cells processed using our culturing conditions are able to undergo expansion without alteration of chromosomal integrity and maintain the expression of signature SC proteins. Our findings collectively support that both human nerve and skin- derived SCs express numerous genes and proteins related to multiple stages of SC development and differentiation states (Jessen and Mirsky, 2005; Krause et al., 2014). Most importantly, we show that adult hSk-SCs can promote axonal outgrowth and initiate myelination of a subset of regenerating axons *in vivo* in a manner comparable to nerve-derived SCs. To our knowledge, this is the first report describing the isolation and extensive *in vivo* characterization of SCs from adult human skin and ultimately their potential relevance for clinical use.

For several years now, the human skin has been explored as a source of SCs to repair the injured nervous system (Toma et al., 2005; Amoh et al., 2009; Liu et al., 2011; Krause et al., 2014). Two studies showed that human SKPs can be isolated and then differentiated into SCs (Wong et al., 2006; Krause et al., 2014), a process that takes more than 8 weeks (Biernaskie et al., 2006) to obtain 3–5 million cells, a cell number appropriate for transplantation in the clinic (Pleasure et al., 1986; Morrissey et al., 1991; Levi and Bunge, 1994a; Guest et al., 2013). In contrast, we found that 3-5 million SCs can be grown directly from the adult human skin (not requiring a skin precursor cell or sphere stage of growth) within five weeks, which allows for expedited application and potentially greater capacity to enhance endogenous repair processes (Fu and Gordon, 1995). Whether these culturing techniques have resulted in the selective growth of dermal mesenchymal-derived SCs or neural crest originating SCs, or both, remain unclear (Widera et al., 2011; Krause et al., 2014). However, from a clinical perspective, given that these cells have the capacity to proliferate in SC media, directly from the adult skin, without the need for reprogramming, we believe they serve as a promising cell type with the capacity to support regeneration following injury.

Following transplantation in nerve injury, we observed robust cell numbers, initiation of a promyelinating phenotype (in ∼55% of transplanted cells), as well as definitive markers of myelination (in ∼4% of transplanted cells) in both adult human nerve and skin-derived SCs (Small et al., 1987; Kamholz et al., 1999). Interestingly, this was in stark contrast to transplanted adult rodent SCs where ∼80% of transplanted cells exhibited mature features of myelin following identical processing as human cells, and transplanted and assessed in the same model (Kumar et al., 2016; Mirfeizi et al., 2017). Such findings suggest that species differences between donor cells versus recipient may play a role in low myelination efficiency of human cells. Nevertheless, others have already shown that expanded adult hN-SCs (like neonatal human *SKP*-derived SCs; Krause et al., 2014) are capable of generating myelin within four to six weeks (Levi and Bunge, 1994a; Levi et al., 1994b). Like us, these studies used multipassaged, expanded human SCs and transplanted these cells into young adult immune-deficient rodents. Strikingly, Levi and colleagues observed that ∼40% of all myelin segments at the center of graft/transplant were derived from human, which is far more than we observed. There are several possibilities for the discrepancy between studies. One obvious difference is the use of prefilled guidance channels where human SCs dominate the graft (Levi et al., 1994b). We used a crush injury model where it is likely that endogenous SCs outcompete human SCs, as hypothesised by others (Levi and Bunge, 1994a). This, however, would not explain the fact that the majority of cells in our study were, in fact, promyelinating and associated with axons. Another possibility is differences between detection approaches between studies. Our study used high resolution imaging and a combination of GFP-labeling to identify transplanted cells with immunohistochemistry to identify axons and multiple myelin-associated proteins; whereas, previous studies have used low resolution imaging and human-specific antibodies alone without axonal markers, or multiple myelin markers to identify transplanted cells. Other contributing factors that may explain low myelination rates, across the board, include *in vitro* expansion (Levi et al., 1994b) and the age of donor SCs. Although we did not detect a difference in myelination (or proliferative capacity and effect on axonal outgrowth) across a 19-year range (27–46 years; data not shown), it is possible that because Levi et al. (1994b) used SCs derived from younger donor patients, largely children and teenagers, their myelination efficiencies were higher than ours. Future studies may need to ascertain whether myelination efficiency is improved in non-human primate models, whether age negatively impacts myelination of skin-derived SCs, or whether there are additional factors that are required to stimulate myelination of human SCs.

It is also plausible that myelination is not the only prerequisite to achieve beneficial outcomes in the clinic. Over three decades ago, it was shown, using several species including human, that transplanted adult nerve-derived SCs have the capacity to sufficiently modulate microenvironments and make them conducive for axon growth and regeneration (Aguayo et al., 1977; Blakemore and Crang, 1985). Since then, several advancements in techniques required for transplants have been made, including the use of matrices to aid in nerve-derived SC survival (Hu et al., 2011; van Neerven et al., 2014), and now, the use of skin-derived SCs to circumvent the invasiveness of cell collection (McKenzie et al., 2006; Biernaskie et al., 2006; Krause et al., 2014). Even with these changes to methodology, researchers have still been able to demonstrate that SCs have the capacity to promote axonal outgrowth (van Neerven et al., 2014). Building on this, our data shows, for the first time, that SCs derived from the adult human skin have the capacity to promote axonal outgrowth, to comparable levels of adult nerve-derived SCs. There are several possible mechanisms underlying these outcomes, including the fact that SCs produce growth factors including FGF-2, BDNF, NT3, and CNTF (Walsh et al., 2009; Allodi et al., 2014). Irrespective of myelination capacity, the inherent capacity to support axonal growth could be sufficient impetus to supplement the chronically denervated distal nerve segment to prevent misdirection and enhance reinnervation of target organs. In addition, we have also shown that transplanted rodent adult skin-derived SCs have a remarkable capacity to enhance debris clearance (Khuong et al., 2014; Stratton et al., 2016). Taken together, it is likely that transplanted adult human SCs function in multiple ways to enhance functional outcomes.

In summary, we demonstrated that skin-derived SCs can be isolated directly from adult human skin and exhibit the capacity to support axonal outgrowth and, to some extent, myelinate axons. Importantly, skin-derived SCs appeared phenotypically indistinguishable to SCs isolated from nerves of the same human subject. Future studies using relevant animal models will be required to determine whether these cells are able to support meaningful behavioural recovery of function following nervous system injury. Given that nerve-derived SCs have shown marked benefit in models of both nerve and spinal cord injury, skin-derived SCs may provide a highly valuable and easily accessible source of autologous myelinating glia to improve outcomes from various neurologic injuries.

## Synthesis

Reviewing Editor: Alfonso Araque, University of Minnesota Decisions are customarily a result of the Reviewing Editor and the peer reviewers coming together and discussing their recommendations until a consensus is reached. When revisions are invited, a fact-based synthesis statement explaining their decision and outlining what is needed to prepare a revision will be listed below. The following reviewer(s) agreed to reveal their identity: David Bennett, Lucia Notterpek.

The reviewers have found merit and interest on the findings reported. I concur with the reviewers' comments expressing the interest of the study. However, they also expressed some concerns about several issues that need to be addressed and clarified. I also consider pertinent the concerns expressed. I believe that addressing those concerns, which will definitely improve the manuscript, is necessarily required to firmly validate the results and support the conclusions.

Specific comments of the reviewers:

Reviewer 1.

General comment:

The generation of human Schwann cells form an adult skin biopsy is certainly a significant advance. The extent of this advance depends on the purity and functionality of these cells. Generally methods are reasonable although there are a number of issues that need to be addressed. The authors repeatedly mention the importance of Schwann cell for functional repair following injury. They show proof of concept that these human cells can remyelinate a small proportion of regenerating axons following crush injury of the mouse. The significance of the paper would have been greatly enhanced had the authors shown improved functional recovery using these cells but it appears that this was not assessed (and group sizes were small).

Major comments:

• The markers used to indicate purity of Schwann cells generated by this culture protocol are Sox10 and nestin. These are not specific to Schwann cells and are expressed widely by the neural crest lineage/progenitors. In figure 4 S100 immunostainning is shown, this is a more specific marker of SCs what propotion of cells generated by this protocol express S100 and have the authors explicitly tested for other neural crest derivatives?

• There are no methods for EM or immunogold labelling these must be added and although this is a difficult technique the images are not very convincing.

Minor comments:

• References are missing from intro - lines 44 and 55 specifically.

• The % of GFP transduction should be mentioned, are all cells transduced? It would be useful for readers to know how amenable human Schwann cells are to viral transduction.

• Is a 2x2cm piece of skin really a ‘clinically relevant sample size’ as suggested? This is actually a large sample for instance compared to diagnostic biopsies. Needs rethinking.

• Figure 4 legend, lines 726 and 727 are incorrect - should be ‘promyelinating and mature myelinating stages’.

• Many DAPI positive nuclei are still present in the DRG explant images (away from the ganglion itself), which makes me question whether endogenous Schwann cells have all been killed by the araC treatment. Co-staining with a Schwann cell marker could address this.

• Fig. 6 - Not at all convinced by the Nav staining - to me this doesn't show a node. Additional staining of the para/juxtaparanode would be required to show a node properly such as Caspr.

Reviewer 2.

The article titled “Isolation and characterization of myelinating Schwann cells from adult human skin” details a procedure for the generation of human Schwann cells from a 2X2 cm piece of skin. The authors provide detailed methodologies and thorough characterization of the isolated Schwann cells with regards to their gene and protein expression profile via RT-PCR and immunostaining, respectively. These human skin-derived Schwann cells express the appropriate markers of Schwann cells at various stages of maturation; and secrete factors that promote neurite outgrowth in culture. In a sciatic nerve crush model, the human skin-derived Schwann cells ensheath regenerating axons and show limited ability to produce myelin proteins. While reproducible methodologies for the generation of human donor-derived Schwann cells is important for the nerve repair/regeneration field, the current manuscript provides limited advancement over already published procedures. In addition, the data in support of the myelination capacity of the isolated cells, which is the major claim and novelty of the current study, has weaknesses.

1) Methods: While it is stated that cell isolation from 4 donors of various ages was performed, it is unclear whether the data shown was obtained with cells from several of the individuals, or just one, and which one? Are there differences in the myelination capacity of the cells from the various donors? Specifically, discussing donor age (ranges from 27-46 yrs. old) as a potential variable, or the lack of, would be important particularly with regards to the functional experiments (neurite outgrowth and myelination).

2) Methods: It would be informative to show the mitotic potential (BrdU uptake assay or something similar) of the cells and to determine how much they could be expanded before loosing their Schwann cell characteristics. Again, donor age should be examined in these experiments as a potential variable.

3) Results: It would strengthen the manuscript if the protein work was supported by Western blots when possible rather than just immunostaining. Data on the expression of P0 and MAG, and the lack of fibroblast proteins such as fibronectin would be helpful on the non-myelinating cultures.

4) Fig 6: The data in support of the myelination capacity of the human Schwann cells is weak. Antibodies to human nuclear antigen (HNA) in combination with MBP should be used to confirm by a second method the human origin of the myelin producing cells in the transplantation paradigm.

5) Fig 6: The EM panel is low in quality and one cannot fully visualize compact myelin. A temporal study of the transplanted cells during nerve regeneration would be beneficial. In addition, testing the cells in the in vitro DRG myelination assay would strengthen the point on the myelination capacity of the skin-derived human Schwann cells.
